# Culture-independent discovery of a novel thermotolerant lipase and its producer from mesophilic anaerobic digestion sludge

**DOI:** 10.1007/s00253-025-13674-0

**Published:** 2025-12-24

**Authors:** Riku Sakurai, Yasuhiro Fukuda, Chika Tada

**Affiliations:** 1 Laboratory of Sustainable Animal Environment, Graduate School of Agricultural Science, Tohoku University, Osaki, Miyagi Japan; 2 Japan Society for the Promotion of Science, Chiyoda-Ku, Tokyo, Japan; 3 Biomanufacturing Process Research Center, National Institute of Advanced Industrial Science and Technology (AIST), Tsukuba, Ibaraki Japan

**Keywords:** Anaerobic digestion, Lipids, Zymography, Metagenomics, Lipase

## Abstract

Anaerobic digestion of lipid-rich wastes holds significant potential for enhanced biomethane production, due to the high energy density of lipids. However, to fully harness this potential, a deeper understanding of lipolytic microorganisms is essential, as key microbial players involved in lipid hydrolysis remain largely unidentified. In this study, we employed an integrated approach combining zymography, metaproteomics, and metagenomics to identify the lipolytic microorganisms from anaerobic digester sludge. This activity-based strategy identified a novel lipase distantly related to known lipases. Besides, although this lipase originates from a mesophilic environment, it exhibited unexpected extremophilic-like properties, with maximal activity at 97.5 °C and pH 11. We further reconstructed a metagenome-assembled genome encoding this lipase and demonstrated that it likely represents a novel genus closely related to *Candidatus* Scatomorpha. Metabolic reconstruction suggested that this bacterium hydrolyzes extracellular lipids and utilizes the resulting hydrolysate, glycerol, to produce lactate and ethanol. Habitat analysis revealed that this bacterium is specifically detected in anaerobic digesters, particularly those processing lipid-rich waste. These findings highlight the pivotal role of this bacterium in anaerobic lipid degradation.

**Supplementary Information:**

The online version contains supplementary material available at 10.1007/s00253-025-13674-0.

## Introduction

Anaerobic digestion is an attractive biotechnology for converting organic wastes into the renewable energy source, biomethane. Lipids are commonly found in various types of wastewaters, including those from edible oil producers, food processing industries, and slaughterhouses (Salama et al. 2019). Lipids exhibit a significantly higher theoretical methane yield (1.0 m^3^ CH₄ kg⁻^1^) than carbohydrates (0.42 m^3^ CH₄ kg⁻^1^) and proteins (0.63 m^3^ CH₄ kg⁻^1^) (Salama et al. 2019), underscoring their potential as highly efficient energy sources. Indeed, co-digestion strategies that incorporate lipid-rich wastes have been shown to markedly enhance biogas production and improve the overall economic viability of anaerobic digestion systems (Iskander et al. 2021).

During anaerobic digestion, lipids are first hydrolyzed by lipase-secreting microorganisms into long-chain fatty acids and glycerol or other compounds (Jenkins 1993; Arndt et al. 2013; Holohan et al. 2022). These hydrolysates are then further degraded to acetate, hydrogen, and carbon dioxide by acidogenic bacteria (Muyzer and Stams 2008; Pelikan et al. 2021; Holohan et al. 2022). Finally, methanogenic archaea convert these products to methane (Lyu et al. 2018). Among these sequential steps, the initial hydrolysis by lipase-secreting microorganisms is often the rate-limiting step and strongly influences the overall process kinetics (Masse et al. 2003; Pascale et al. 2018; Li and Shimizu 2021).

Despite its critical role, surprisingly little is known about the microorganisms responsible for lipid hydrolysis. Current evidence only suggests that members of the Bacteroidetes, Proteobacteria, and Firmicutes phyla may play a role in this process (Petropoulos et al. 2018; Salama et al. 2019). Bashiri et al. (2022) employed metagenomics and metaproteomics to identify key lipolytic microorganisms; however, they concluded that such microorganisms were difficult to identify using these approaches. Notably, they observed that the number of lipases annotated in the metagenomes was significantly lower than that of other extracellular hydrolytic enzymes. These findings suggest that proteins with lipolytic activity in anaerobic systems may be only distantly related to known lipases, making their identification by sequence homology particularly challenging. More broadly, beyond anaerobic digestion, only 15 lipases have been isolated and characterized from anaerobic microorganisms (Yu et al. 2010; Henderson 1971; Privé et al. 2013; Petersen and Daniel 2006; Salameh and Wiegel 2007; Sarada and Joseph 1992; Royter et al. 2009; Behere et al. 2002; Biundo et al. 2016; Kim et al. 1996). These observations underscore the considerable difficulty in identifying functional lipolytic microorganisms in anaerobic ecosystems using conventional cultivation or sequence-based approaches.

In this study, we adopted a functional metaproteomic workflow inspired by that of Sukul et al. (2017). They integrated zymography with metaproteomics and metagenomics to directly identify esterases that were active against the fluorogenic substrate 4-methylumbelliferyl butyrate in soil samples (Sukul et al. 2017). Building on this methodology, we performed a lipase-specific screening with the fluorogenic substrate 4-methylumbelliferyl oleate. Our approach enables the a priori identification of proteins with activity against long-chain fatty acid esters (i.e., lipases (Brockerhoff and Jensen 1974)) in the metaproteome of the sludge collected from an anaerobic digester.

## Materials and methods

### Sludge preparation and DNA/protein extraction

Anaerobic digester sludge was collected from a lipids-rich full-scale mesophilic food waste treatment plant (Tokyo, Japan), which contained an average of 1650 mg L^−1^ lipids and long-chain fatty acids during a 9-month monitoring period. Genomic DNA was extracted using the Fast DNA®SPIN Kit for Soil (MP Biomedicals). The DNA concentration was measured with a QuantiFluor dsDNA System and a Quantus Fluorometer (Promega). DNA quality was assessed using the 5200 Fragment Analyzer System and Agilent HS Genomic DNA 50 kb Kit (Agilent Technologies).

Total protein was extracted via bead-beating. Equal volumes (450 µl each) of the sludge and RIPA buffer (Cayman Chemical) were placed in a Lysing Matrix E tube (MP Biomedicals) and homogenized using a Micro Smash (Tomy) at 3000 rpm for 120 s, repeated once. After centrifugation (12,000 × g, 10 min), the supernatant (500 µl) was mixed with 50 µl ice-cold trichloroacetic acid, incubated for 30 min, and centrifuged (14,000 × g, 10 min). The pellet was washed twice with acetone at −20 °C, air-dried, and resuspended in 450 µl of Ez Apply 2D Kit Solution 2 (ATTO) using a HANDY Sonic UR-21P (Tomy). After adding 40 µl of Solution 2–2 and incubating for 10 min, the final protein solution was stored at −80 °C. Procedures were performed on ice unless noted otherwise.

### 2D gel electrophoresis and zymography

Fifteen microliters of the protein solution and 10 µl of the overlay solution from the Ez Apply 2D Kit (ATTO) were loaded onto an Ager GEL A-M38 (ATTO). Isoelectric focusing was carried out at 300 V for 210 min using the WSE-1510 DiscRun Ace (ATTO). The agar gel was subsequently shaken in 2.5% trichloroacetic acid solution and then washed with distilled water. Prior to SDS‒PAGE, the agar gel was equilibrated for 10 min in buffer composed of 50 mM Tris (pH 6.8), 2% SDS, and 0.01% bromophenol blue (BPB). The agar gel was placed onto an 8% SDS‒polyacrylamide gel containing 100 µM 4-methylumbelliferyl oleate dissolved in 25 µl of DMSO. Electrophoresis was carried out at 200 V for 60 min in running buffer composed of 1% SDS, 1.92 M glycine, and 250 mM Tris.

After electrophoresis, the proteins were renatured by gently agitating the gel in a 2.5% Triton X-100 solution for 60 min. Afterward, the gel was washed with distilled water and incubated at 35 °C in 50 mM phosphate buffer (pH 7). Lipase activity was detected by the hydrolysis of 4-methylumbelliferyl oleate, producing fluorescent 4-methylumbelliferone under UV light. Protein quantity was estimated using a Silver Stain MS Kit (APRO Science Group).

### Mass spectrometry analysis

The protein spots with lipase activities were excised from the gel and sent to Japan Proteomics Co., Ltd. (Sendai, Japan) for mass spectrometry analysis. Briefly, after in-gel digestion, the resulting peptides were analyzed via nanoLC‒MS/MS. The generated mass spectrometry data were searched against a metagenomics-derived database using the Mascot program (Matrix Science).

### Metagenomic analysis

Sequencing was performed using a PacBio Sequel IIe (Pacific Biosciences) and a DNBSEQ-G400 (MGI Tech) at Bioengineering Lab. Co., Ltd. (Kanagawa, Japan) (Sakurai et al. 2024). For PacBio sequencing, the library was prepared with the SMRTbell Express Template Prep Kit 2.0, and the sequencing polymerase was bound using the Binding Kit 2.2 (Pacific Biosciences). After sequencing, adaptors were trimmed with SMRT Link v. 10.1.0. HiFi reads were generated using pancake with KSW2 (Pohjola et al. 2023), yielding 157,984 reads (average: 6957 bp). For DNBSEQ sequencing, the library was constructed with the MGIEasy FS DNA library prep set, and quality was confirmed using the Fragment Analyzer and dsDNA 915 Reagent Kit (Agilent). Circular DNA was prepared with the MGIEasy Circularization Kit, followed by DNB generation using the DNBSEQ-G400RS High-throughput Sequencing Kit. Paired-end (2 × 200 bp) sequencing was performed, and reads were processed using Trimmomatic v. 0.39 (Bolger et al. 2014) and FastQC v. 0.11.9 (https://www.bioinformatics.babraham.ac.uk/projects/fastqc/).

Hybrid assembly was performed by metaSPAdes v. 3.10.1 (Antipov et al. 2016), and the HiFi reads were separately assembled by hifiasm_meta v. 0.3.1 (Feng et al. 2022). The assemblies were merged using quickmerge v. 0.3 (Chakraborty et al. 2016) and scaffolded with ntLink (Coombe et al. 2023). Coverage was calculated by mapping DNBseq reads with Bowtie2 v. 2.4.1 (Langmead and Salzberg 2012). Binning was performed using SemiBin2 v. 1.5.1 with the built-in model “wastewater” (Pan et al. 2023). Potential contamination in the metagenome-assembled genome (MAG) was detected and removed via the MDMcleaner pipeline v. 0.8.7 (Vollmers et al. 2022), and evaluated by CheckM2 (Chklovski et al. 2023). Coding sequences were predicted with Prokka v. 1.14.6 (Seemann 2014), and the resulting protein database was used for the Mascot search. These analyses were performed with default parameters unless otherwise mentioned.

MAG taxonomy was assigned using GTDB-Tk v. 2.3.2 (Chaumeil et al. 2022). The percentage of conserved proteins (POCP) was calculated by the POCP-nf pipeline (Hölzer 2024). Metabolic reconstruction was performed by BlastKOALA and the Integrated Microbial Genomes & Microbiomes system (IMG/MER: https://img.jgi.doe.gov/cgi-bin/mer/) (Chen et al. 2023). The habitability and distribution of the target microorganism were investigated via the IMNGS platform (Lagkouvardos et al. 2016) and ProkAtlas (Mise and Iwasaki 2020) tool, with a sequence similarity threshold of 99%.

### Heterologous expression of putative lipases

The target gene was amplified by PCR using the extracted DNA from the sludge as a template. The primer set used here contained recognition sites for *BamHI* and *NotI*. The forward primer was 5′- TAATTCGGATCCGGGAAGCTCTCCGGCAAAGTCC −3′, and the reverse primer was 5′- TCGAGTGCGGCCGCTTCAATGGGAGAGATGTCGGCA −3′. PCR was carried out using PrimeSTAR® Max DNA Polymerase (Takara Bio), and the resulting amplicons were purified using a Monarch DNA Gel Extraction Kit (NEB). Both the purified PCR products and the pET22(b)+ vector were digested with BamHI HF and NotI HF in CutSmart Buffer (NEB) and ligated using the DNA Ligation Kit (Takara Bio). Clones were sequence-verified via a DS3000 compact CE sequencer (HITACHI).

The recombinant vector was introduced into *E. coli* BL21 by electroporation. BL21 cells were preincubated overnight in LB medium with 100 μg ml⁻^1^ ampicillin, then transferred to fresh LB medium and grown to OD600 = 0.5–0.7. IPTG (2 mM) was added, and cells were incubated for 2.5 h before harvesting by centrifugation. The pellet was resuspended in 20 mM sodium phosphate buffer (pH 7.4) with 0.5 M NaCl and 55 mM imidazole, then sonicated (HANDY Sonic UR-21P, Tomy). The target protein was purified using HisTrap™ HP (Cytiva), and buffer-exchanged to 50 mM sodium phosphate (pH 7.0) with 62.5 mM NaCl using an Amicon Ultra 15 mL filter (3 kDa MWCO, Merck Millipore).

### In silico characterization of hp2-1

Comparison of the hp2-1 sequence with representatives of known lipolytic enzyme families (35 families and 11 true subfamilies) was performed using Lipase_reclassification (Hitch and Clavel 2019). Functional prediction was performed using InterPro (https://www.ebi.ac.uk/interpro/) (Blum et al. 2024) with the default settings. The signal peptide sequence and its cleavage sites were predicted using the SignalP-6.0 server in slow mode (https://services.healthtech.dtu.dk/services/SignalP-6.0/) (Teufel et al. 2022).

AlphaFold3 was used to predict the three-dimensional structure of the hp2-1 protein on the basis of its amino acid sequence (Abramson et al. 2024). The results were visualized and analyzed by PyMOL (https://github.com/schrodinger/pymol-open-source). The structural homology search was performed by Protein structure comparison service PDBeFold (http://www.ebi.ac.uk/msd-srv/ssm) (Krissinel and Henrick 2004).

### Substrate specificity

The chain length specificity was evaluated by a *p*-nitrophenyl acyl ester assay using various alkyl chain lengths from C4 to C18 (C6: Tokyo Chemical Industry Co., Ltd.; C16: FUJIFILM Wako Pure Chemical Co.; and others: Sigma‒Aldrich). Each substrate was prepared as a 20 mM stock solution in 99% ethanol and stored at −20 °C in the dark. The reaction was carried out by adding 10 µl of the substrate solution, 180 µl of assay buffer (50 mM sodium phosphate buffer (pH 7.0) supplemented with 1 mg ml^−1^ gum arabic and 0.4% Triton X-100), and 10 µl of 0.32 mg ml^−1^ purified protein solution to the 96-well plates. The absorbance was measured at *λ* = 410 nm with a DS PHARMA BIOMEDICAL (Bio-Tec) microplate reader after gently shaking for 15 min at 50 °C. The positional specificity was evaluated via the use of triolein, 1,3-diolein, and monoolein (Sigma‒Aldrich). Each substrate was prepared as a 10 mM stock solution in 99% 2-propanol and stored at −20 °C in the dark. The reaction was carried out by mixing 5 µl of the substrate solution, 90 µl of assay buffer, and 5 µl of 0.057 mg ml^−1^ purified protein solution in 1.5 ml centrifuge tubes, followed by incubation at 50 °C for 45 min on a heating block. The released glycerol was measured using a commercial glycerol assay kit according to the manufacturer’s instructions (Sigma‒Aldrich). All values were determined in triplicate and corrected for autohydrolysis by blank (without enzyme) subtraction.

### Optimal temperature and pH

Lipase activity was assessed by a *p*-nitrophenyl palmitate assay at temperatures ranging from 20 °C to 97.5 °C. The reaction was carried out by mixing 10 µl of the substrate stock, 180 µl of assay buffer, and 10 µl of the 0.26 mg ml^−1^ purified protein solution in 1.5 ml centrifuge tubes, followed by incubation on a heating block for 1.5 h. The reaction was terminated by placing the samples on ice blocks and adding NaOH at a final concentration of 5 mM. The reaction mixture was subsequently transferred to 96-well plates, and the absorbance was measured at *λ* = 410 nm with a DS PHARMA BIOMEDICAL (Bio-Tec) microplate reader. The assay buffer was prewarmed to the desired reaction temperature. Protein thermal stability was assessed using differential scanning fluorimetry (DSF). Purified protein (18  µL at 0.001 mg mL⁻^1^) was mixed with 2  µL of 1000 × SYPRO Orange dye (Sigma-Aldrich) in each well of a qPCR plate and vortexed. Fluorescence measurements were performed on a Bio-Rad CFX Connect Real-Time System, with a temperature scan from 30 °C to 99 °C at a ramp rate of 0.5 °C every 10 s.

The optimal pH of hp2-1 was evaluated by measuring its activity against monoolein in the following buffers: glycine–HCl (pH 3), acetate (pH 4, 5), sodium phosphate (pH 6, 7), Tris–HCl (pH 8, 9), and carbonate-bicarbonate (pH 10, 11). All buffers were prepared at 50 mM and contained 1 mg ml^−1^ gum arabic and 0.4% Triton X-100. The reaction was carried out as described for the positional specificity assay, with monoolein used as the substrate. All measurements were performed in triplicate, and values were corrected for autohydrolysis by subtracting blanks (reactions without enzyme).

## Results

### Identification of lipolytic proteins in the anaerobic digester sludge

**Fig. 1:**
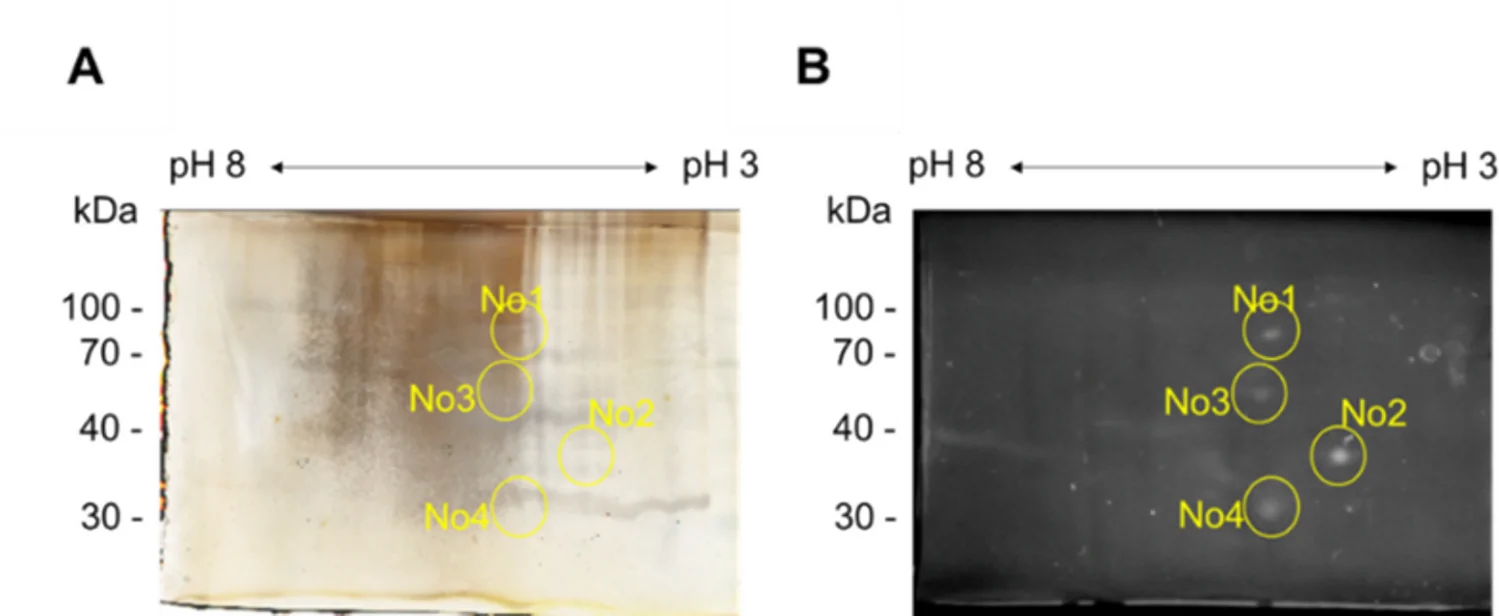
Functional metaproteomic analysis of anaerobic digester sludge. **A** 2D gel electrophoresis of the anaerobic digester sample, visualized by silver stain. **B** In-gel activity assay (zymography) for detecting lipase

**Fig. 2:**
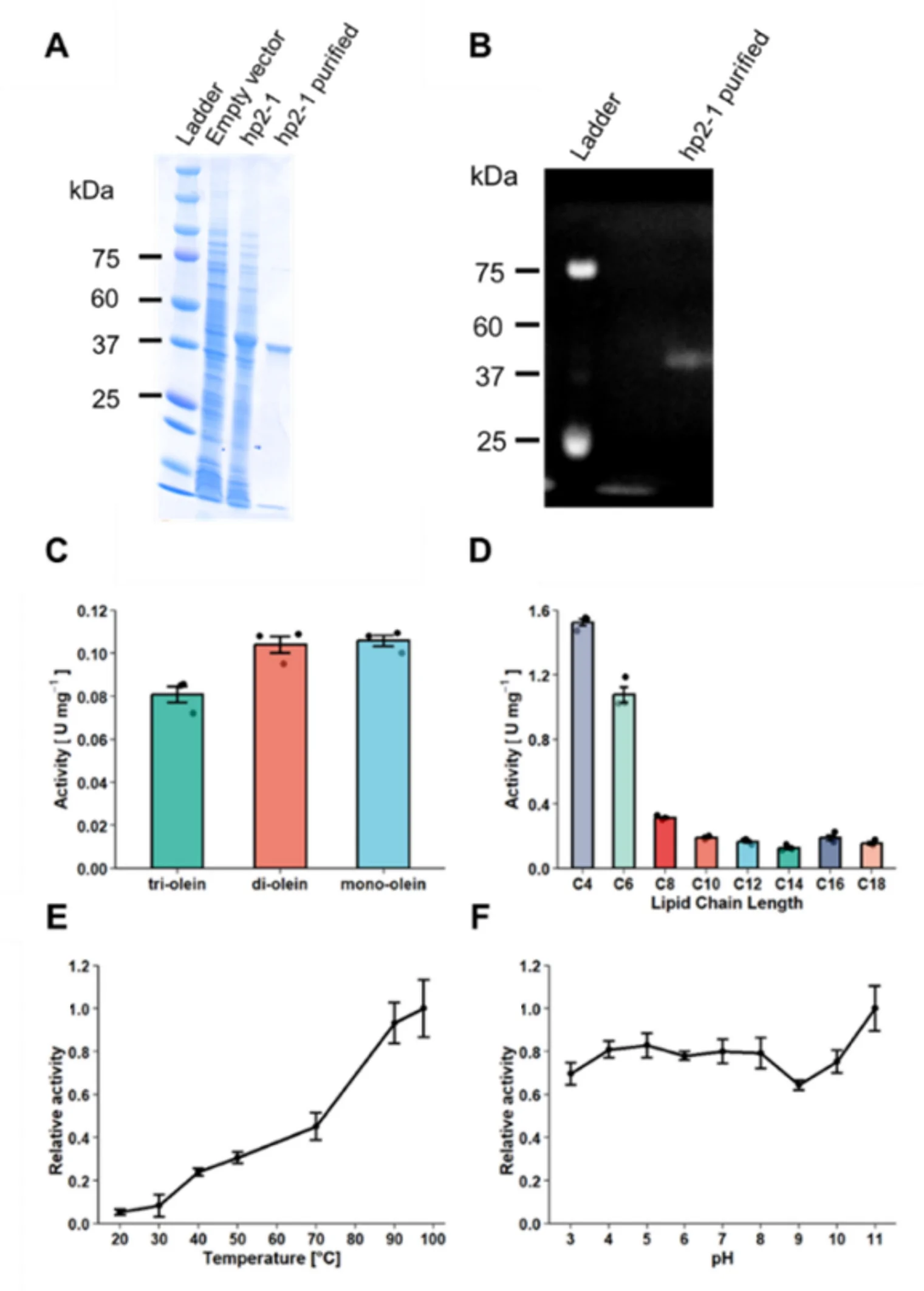
Heterologous expression, purification, and characterization of hp2-1. **A** Visualization of the protein content by Coomassie blue staining after SDS-PAGE. **B** Lipid hydrolysis activity of purified hp2-1 detected by in-gel zymography. **C** Positional specificity and **D** chain length selectivity of hp2-1. Chain length selectivity was assessed using *p*-nitrophenyl compounds with acyl-chain lengths ranging from C4 to C18. **E** Effect of temperature on hp2-1 activity against *p*-nitrophenyl palmitate. **F** Effect of pH on hp2-1 activity against monoolein

The 2D-gel zymography identified four lipolytically active protein spots (Fig. 1), which were excised and analyzed by mass spectrometry. To facilitate protein identification, a metagenome-derived database comprising 146,811 coding sequences from 121 MAGs was constructed. Using this database, several proteins were identified from each spot, except for spot No. 1. Although none of these proteins were annotated as lipases, hypothetical proteins were detected (Table 1). On the basis of this finding, we hypothesized that these hypothetical proteins might have exerted lipolytic activity. Through the heterologous expression experiments, we revealed that the hypothetical protein 2–1 (hp2-1), which was identified in spot No. 2, exhibits lipolytic activity (Fig. 2A, B). For the hypothetical proteins identified from other spots (No. 3 and 4), expression systems could not be established in our experiments. The positions of protein spots on the 2D gel do not always correspond to their theoretical molecular weights, due to factors such as abnormal SDS-binding and electrophoretic behavior (Rath et al. 2009). Therefore, these proteins, including those whose positions do not match the expected molecular weights, may also represent novel lipases and warrant further investigation.

**Table 1:** List of proteins identified from 2D gels by Mascot search

Spot	Protein	MW	Score	Coverage
No. 1	*n.d*			
No. 2	Hypothetical protein (hp2-1)	34,496	154	15
	Membrane lipoprotein TmpC	39,085	89	16
	Hypothetical protein (hp2-2)	21,632	85	17
	Hypothetical protein (hp2-3)	184,767	85	1
	Multiple sugar-binding periplasmic protein SbpA	38,072	73	11
No. 3	Enolase	46,914	84	19
	ATP synthase subunit beta	51,390	75	24
	Hypothetical protein (hp3-1)	26,932	68	11
	ATP synthase subunit beta	51,057	61	9
	Hypothetical protein (hp3-2)	12,998	57	10
	Enolase	31,352	44	4
No. 4	Hypothetical protein (hp4-1)	25,122	78	13
	Butyrate–acetoacetate CoA-transferase subunit B	22,552	58	6
	Hypothetical protein (hp4-2)	30,109	53	5
	Hypothetical protein (hp4-3)	104,853	38	1

Score: Probability-based Mowse score. The ion score is −10*Log(P), where P is the probability that the observed match is a random event

Individual ion scores >33 indicate identity or extensive homology (*p* < 0.05). Protein scores are derived from ion scores as a nonprobabilistic basis for ranking protein hits

### Characterization of hp2-1

The latest classification tool for lipolytic enzymes, Lipase_reclassification, indicated that hp2-1 could not be assigned to any of the current 35 families or 11 subfamilies (Figure S1). Rather, in silico characterization using InterPro suggested that hp2-1 was categorized as ABC transporter substrate-binding proteins (SBPs) (Figure S2). SBPs typically bind to specific extracellular substrates and deliver them to the membrane-associated permease (Binet et al. 1997). To our knowledge, no substrate-binding proteins have been demonstrated to catalyze hydrolysis. SignalP 6.0 analysis predicted a Sec/SPII-type signal peptide at the N-terminal region of hp2-1 (Figure S3), suggesting secretion into the extracellular space via the Sec translocon, as is often observed in bacterial SBPs (Renier et al. 2012). Please note that the coding sequence for the first 20 amino acid residues at the N-terminus, including the signal peptide, was excluded from the expression construct.

Rather, it appears to have originated from SBP. This raises the question of whether its catalytic mechanism is consistent with those of characterized lipases or represents a distinct mode of hydrolysis. To address this, a *p*-nitrophenyl palmitate assay was conducted in the presence of the lipase-inhibitor, phenylmethylsulfonyl fluoride (PMSF). We found that with the presence of 0.48 mM PMSF, hp2-1 lost its lipolytic activity (Figure S4), suggesting that its catalytic mechanism is similar to that of known lipases. To investigate the enzymatic properties of hp2-1, the positional specificity, chain-length selectivity, optimal temperature, and pH were analyzed (Fig. 2C–F). The positional specificity was tested using tri-, di-, and monoolein as substrates, demonstrating higher activities against diolein and monoolein than that against triolein (TukeyHSD, *p* < 0.02). The chain-length selectivity was evaluated using *p*-nitrophenyl esters with acyl lengths ranging from C4 to C18. hp2-1 exhibited hydrolytic activity against all the *p*-nitrophenyl compounds tested, with the highest activities for C4-8 esters. There were no significant differences in the activity for C10-18 esters (TukeyHSD, *p* > 0.05). Notably, the lipolytic activity of hp2-1 against *p*-nitrophenyl palmitate increased with increasing temperature, peaking at approximately 97.5 °C. DSF indicated that hp2-1 displayed only minor fluctuations in fluorescence across the entire temperature range (30–99 °C), without a distinct unfolding peak (Figure S5). Additionally, hp2-1 showed stable activity across a wide pH range (3 to 11), with the highest activity occurring at pH 11 (TukeyHSD, *p* < 0.02).

### Genome reconstruction of hp2-1-encoding microorganism

**Fig. 3:**
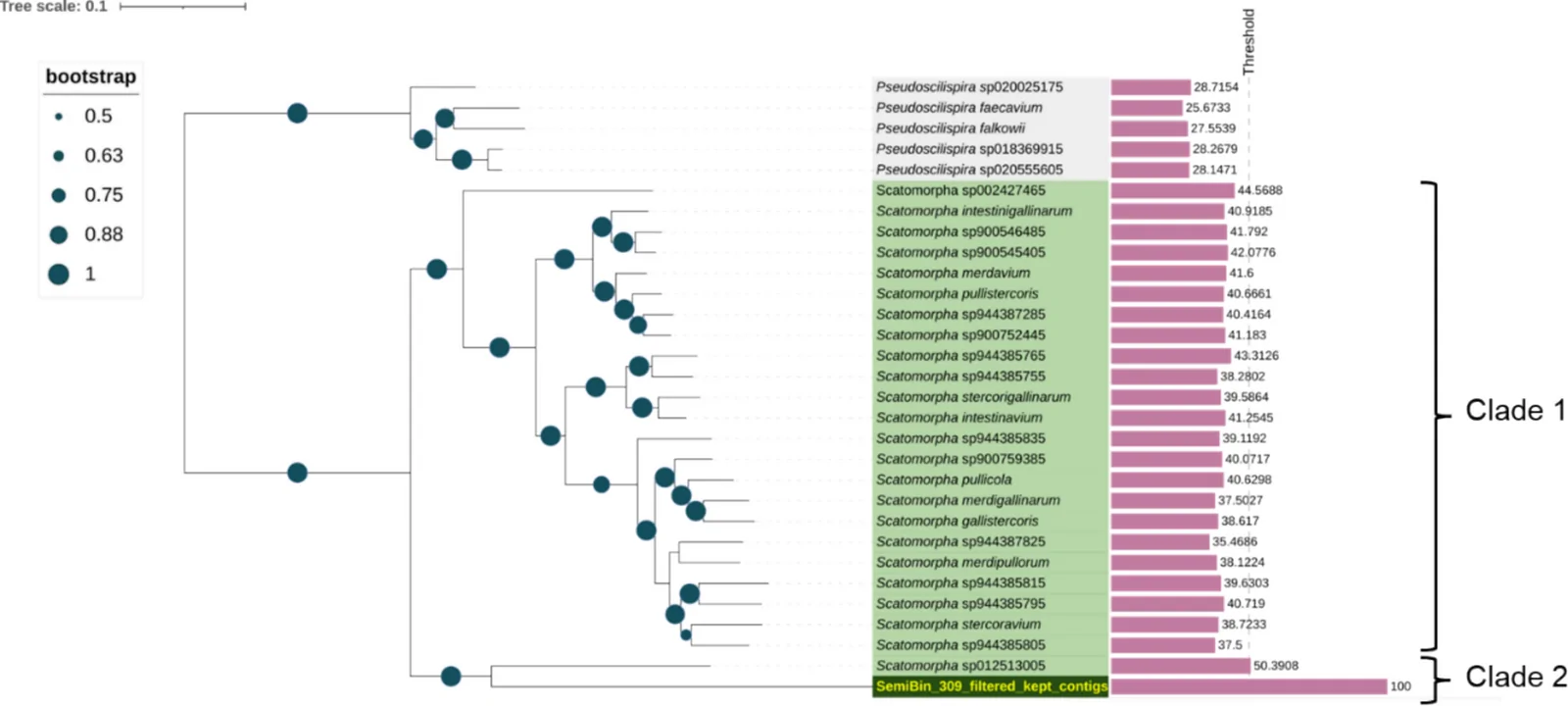
Phylogenetic analysis of SemiBin_309 at the genus level on the basis of concatenated phylogenetic marker genes in GTDBtk v.2.3.2. The GTDB species representatives of the genus *Ca*. Scatomorpha are highlighted in green for clarity. The percentage of conserved proteins (POCP) compared with those in SemiBin_309 are also shown on the right side of the bar plot

The *hp2-1* gene was identified in a reconstructed genome (SemiBin_309), which was taxonomically classified into the genus *Candidatus* (*Ca.*) *Scatomorpha* using GTDB-Tk classify_wf (Chaumeil et al. 2022). The genome comprises 16 contigs with a total size of 2,129,914 bp, containing two 16S rRNA genes, one 23S rRNA gene, and one 5S rRNA gene. The completeness and contamination rates were estimated as 95.9% and 0.81%, respectively. Notably, phylogenetic analysis of SemiBin_309 using GTDBtk denovo_wf (Chaumeil et al. 2022) suggested that *Ca.* Scatomorpha can be divided into two distinct clades (Fig. 3). The percentage of conserved proteins (POCP) between another MAG in ‘Clade 2’ (Scatomorpha sp012513005) and SemiBin_309 was 50.3%, whereas the POCP between MAGs in ‘Clade 1’ and SemiBin_309 ranged from 37.5% to 44.6% (Fig. 3, bar plot). Given that a POCP threshold of 50% has been proposed as a genus boundary for prokaryotic lineages (Qin et al. 2014), ‘Clade 2’ should represent a different genus from the other members of *Ca*. Scatomorpha. We also performed order-level phylogenetic analysis (Figure S6), which supported that ‘Clade 2’ possibly represents a novel genus most closely related to *Ca*. Scatomorpha.

**Fig. 4:**
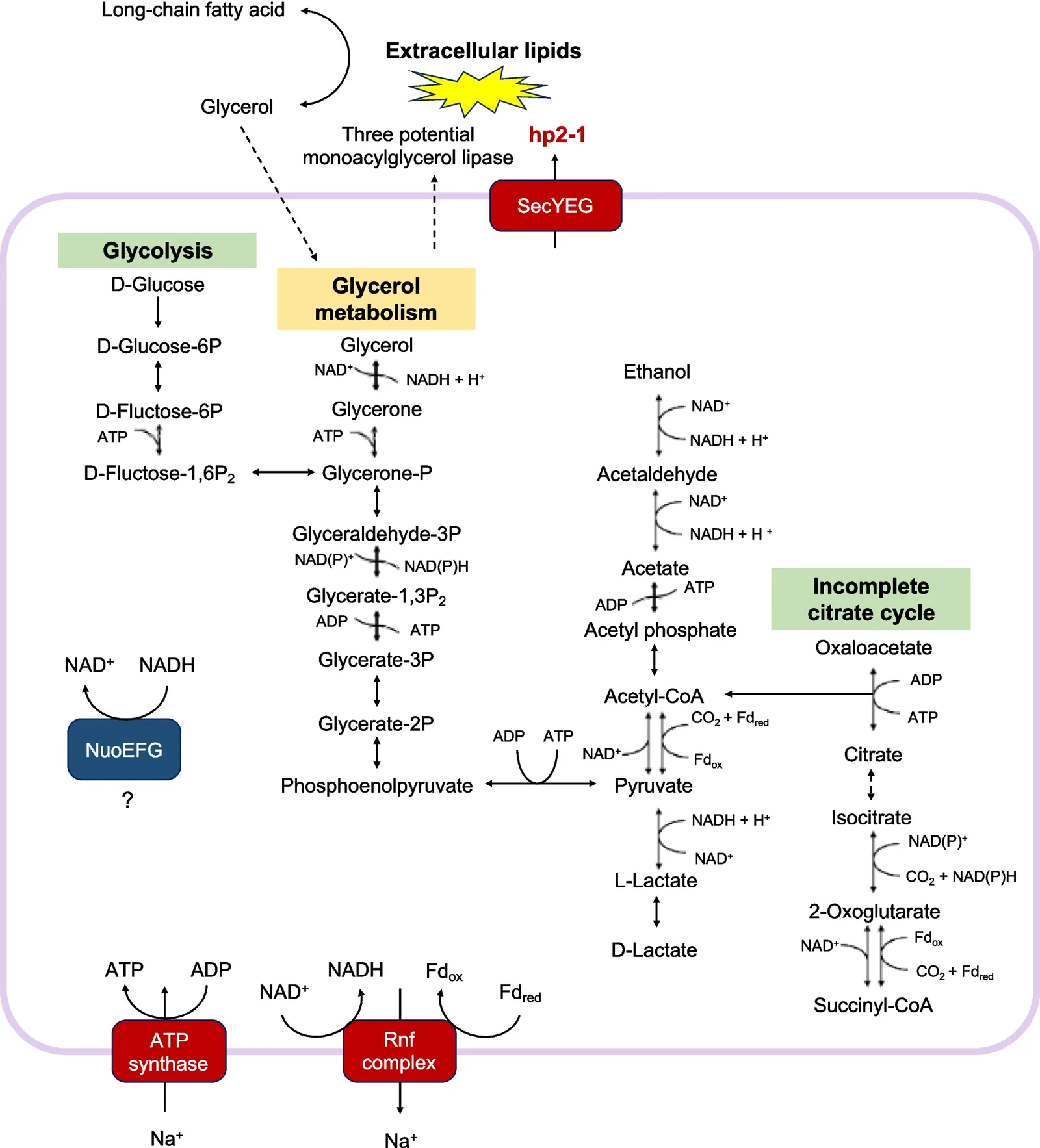
Metabolic reconstruction of hp2-1-secreting bacteria (SemiBin_309). Solid line: detected; dashed line: not detected

The metabolic capabilities of the hp2-1-secreting bacteria are summarized in Fig. 4 (see Source_Data for detail). Notably, in addition to hp2-1, five genes annotated as lipases were identified. Lipase-secreting bacteria typically utilize lipid hydrolysates such as glycerol and long-chain fatty acids (Sarada and Joseph 1992; Svetlitshnyi et al. 1996). Although no genes associated with fatty acid degradation were detected, two distinct glycerol metabolic pathways were discerned. In the first pathway, glycerol is converted into dihydroxyacetone (glycerone) by glycerol dehydrogenase. Dihydroxyacetone is then phosphorylated to dihydroxyacetone phosphate by dihydroxyacetone kinase, allowing it to enter the glycolysis pathway. In the second pathway, glycerol is initially oxidized to D-glyceraldehyde by glycerol dehydrogenase (NADP^+^). D-glyceraldehyde is then oxidized to 2-phospho-D-glycerate by aldehyde dehydrogenase (NAD^+^), subsequently entering the glycolysis pathway. However, glycerol dehydrogenase (NADP^+^) was not identified in our analysis. These findings suggest that this bacterium can utilize glycerol, which is obtained through the secretion of lipases, including hp2-1, as an energy source. In addition to glycerol metabolism, central carbon pathways, including glycolysis, the phosphate acetyltransferase–acetate kinase pathway, and the lactate fermentation pathway, were identified. The tricarboxylic acid (TCA) cycle appeared incomplete, with only the conversion steps for isocitrate, 2-oxoglutarate, and succinyl-CoA present. A primary ATP generation pathway is likely driven by a membrane-bound ATP synthase, which utilizes a transmembrane Na⁺ gradient established by the Rnf complex. The Rnf complex generates a Na⁺ gradient by coupling the reduction of NAD⁺ with the oxidation of reduced ferredoxin (Fd_red_) (Kuhns et al. 2020). In this bacterium, NAD⁺ is inferred to be regenerated through lactate fermentation or ethanol production from acetate. This ethanol synthesis pathway has been identified in the strict anaerobe *Clostridium ljungdahlii*, where it plays a crucial role in maintaining the NADH/NAD^+^ balance (Liu et al. 2020). Additionally, the N module of bacterial Respiratory Complex I (NuoE, NuoF, and NuoG) was identified. A previous study reported that in *E. coli*, this module can form active units independently of other membrane components, enabling NADH oxidation in the soluble fraction (Alkhaldi and Vik 2022). Nuo EFG may also play a role in maintaining the redox balance in this bacterium.

As mentioned above, hp2-1 was predicted to contain Sec/SPII-type signal peptides. In this system, synthesized proteins are transported to the extracellular space by the SecYEG complex and cleaved by signal Peptidase II (*Lsp*). Homologs of the SecYEG complex and Lsp were also identified in the reconstructed MAG, further supporting the in silico prediction that hp2-1 is secreted through the Sec/SPII pathway.

### Habitat analysis of the novel lipolytic bacterium

Thus far, the characteristics of the novel lipase hp2-1 and the bacterium that encodes it have been elucidated. To further understand the ecological role of this bacterium, habitat analysis was conducted using ProkAtlas (Mise and Iwasaki 2020) and IMNGS (Lagkouvardos et al. 2016). The ProkAtlas platform utilizes 16S rRNA information derived from deposited shotgun metagenomic data, whereas IMNGS employs 16S rRNA sequences from deposited amplicon sequencing data. The analysis by ProkAtlas indicated that the 16S rRNA sequence of the hp2-1-encoding bacterium was detected exclusively in biogas fermenters, with a habitat preference score of 100%. This finding suggests that this bacterium is specifically adapted to anaerobic wastewater treatment systems. IMNGS analysis further identified the primary sources where the 16S rRNA sequence of the hp2-1-encoding bacterium was predominantly detected (Table 2). Notably, it was abundant in anaerobic digesters treating sugar beet silage, maize silage, cheese whey, and food waste, comprising up to 18.6% of the microbial community in some samples. All these reactors were operated under mesophilic conditions, suggesting that the hp2-1-secreting bacterium is mesophilic.

**Table 2:** Source and relative abundance of the hp2-1-secreting bacterium identified via IMNGS analysis. The full-length 16S rRNA sequence Identified from SemiBin_309 was used as the query. A similarity threshold of 99% and a minimum alignment length of 200 bp were applied

#SampleID	Description	Relative abundance (%)	Substrates	Operation conditions
ERR695523	biogas fermenter metagenome	18.6	Sugar beet silage maize silage	Mesophilic, CSTR
SRR8138777	anaerobic digester metagenome	17.7	Cheese whey	Mesophilic, CSTR
ERR695522	biogas fermenter metagenome	16.3	Sugar beet silage maize silage	Mesophilic, CSTR
SRR8138778	anaerobic digester metagenome	7.1	Cheese whey	Mesophilic, CSTR
ERR579105	anaerobic digester metagenome	5.0	Cattle or swine manures	N. D
SRR8138779	anaerobic digester metagenome	2.9	Cheese whey	Mesophilic, CSTR
ERR1670653	metagenome	2.4	N. D	N. D
ERR1670655	metagenome	2.4	N. D	N. D
ERR1670654	metagenome	2.4	N. D	N. D
SRR1723000	anaerobic digester metagenome	2.3	N. D	N. D
SRR3218648	biogas fermenter metagenome	1.9	Maize silage	N. D
SRR2917881	anaerobic digester metagenome	1.7	Food waste	Mesophilic, CSTR
SRR8138780	anaerobic digester metagenome	1.5	Cheese whey	Mesophilic, CSTR
SRR2025016	fermentation metagenome	1.5	Dairy sewage sludge, fruit waste, corn silage and grain decoction	N. D
SRR8529830	biogas fermenter metagenome	1.3	N. D	N. D
ERR1670649	metagenome	1.1	N. D	N. D

## Discussion

The present study identified the novel lipase hp2-1 and its encoding bacterium, through an integrated approach that combined zymography, metaproteomics, and metagenomics. While this approach has some limitations, such as the difficulty of isolating all proteins from environmental samples and refolding them correctly (Almeida et al. 2020), it provides a significant advantage by enabling a priori screening—allowing for the direct identification of specific enzymes and their producers from environmental samples. Broader application of this method could advance our understanding of key lipolytic microorganisms involved in anaerobic digestion, thereby contributing to the optimization of lipid-rich waste degradation processes.

The most remarkable feature of hp2-1 would be its exceptional extremophilic-like properties, with optimal activity at approximately 97.5 °C or pH 11 (Fig. 2E, F). To date, only three lipases with optimal temperatures above 90 °C have been reported. Among them, two are produced by the anaerobic bacterium *Thermosyntropha lipolytica,* with optimal activity at 96 °C (Salameh and Wiegel 2007), and one by the aerobic bacterium *Thermomicrobium roseum,* active at 90 °C (Fang et al. 2021). Unlike these enzymes, hp2-1 demonstrates a remarkably broad temperature range, maintaining activity from 20 °C to 97.5 °C. Furthermore, its stable activity across a wide pH range (pH 3–11) highlights its potential for industrial applications beyond those of conventional lipases.

The metabolic reconstruction of the hp2-1-secreting bacterium indicated that this bacterium encodes an additional five potential lipases and could generate ATP by degrading glycerol to acetate. Habitat analysis supported this observation, suggesting that the 16S rRNA sequence of this bacterium is abundant (as high as 18.6%) in anaerobic digesters used to treat cheese whey, food waste, and silage-related compounds. Cheese whey wastewater and food waste are known to contain sufficient fat to cause sludge floatation (*i.e*., scum formation) (Carvalho et al. 2013; Xu et al. 2018). The sludge used in this study was also collected from a lipid-rich food waste treatment plant, as mentioned in the Materials and Methods section. Taken together, these results highlight the cardinal roles of the hp2-1-secreting bacterium in anaerobic lipid degradation.

In conclusion, we identified a novel lipolytic enzyme, hp2-1, and its encoding bacterium from an anaerobic digester. Phylogenetic analysis and POCP values suggest that this bacterium, together with *Ca*. Scatomorpha sp012513005, may represent a new genus yet to be formally classified. Notably, hp2-1 is only distantly related to known lipases, highlighting the limitations of sequence-based approaches for detecting such novel enzymes. This finding supports a previous observation by Bashiri et al. (2022) that a large part of lipolytic enzymes may share low sequence similarity with known lipases. We hope that this study serves as a model for future investigations into the microbial ecology of lipid hydrolysis in anaerobic digestion, with the goal of optimizing methane production from lipid-rich wastes. Furthermore, the unexpected thermostability and broad pH tolerance of hp2-1 highlight its strong potential for various biotechnological applications beyond anaerobic digestion, particularly in industrial processes operating under extreme temperature or pH conditions.

## Supplementary Information

Below is the link to the electronic supplementary material.

ESM1 (DOCX 996 KB)

ESM2 (XLSX 16.4 MB)

## Data Availability

The sequence data are deposited under BioSample accession number SAMD00662589.
