# Supplementary material for: Culture-independent discovery of a novel thermotolerant lipase and its producer from mesophilic anaerobic digestion sludge
Source: Appl Microbiol Biotechnol. 2025 Dec 24;109:283. doi: 10.1007/s00253-025-13674-0 (PMC12740960; doi:10.1007/s00253-025-13674-0)
Supplement: Supplementary file 1 [file 253_2025_13674_MOESM1_ESM.docx]

**Supporting Information:**

Culture-independent discovery of a novel thermotolerant lipase and its producer from mesophilic anaerobic digestion sludge

Riku Sakurai ^1, 2, 3^, Yasuhiro Fukuda ^1^, Chika Tada ^1*^.

^1^ Laboratory of Sustainable Animal Environment, Graduate School of Agricultural Science,Tohoku University, Osaki, Miyagi, Japan

^2^ Japan Society for the Promotion of Science, Chiyoda-ku, Tokyo, Japan

^3^ Biomanufacturing Process Research Center, National Institute of Advanced Industrial Science and Technology (AIST), Tsukuba, Ibaraki, Japan

*Chika Tada

Email: [chika.tada.e1@tohoku.ac.jp](mailto:chika.tada.e1@tohoku.ac.jp)

Figure S1. Classification of hp2-1 into families and subfamilies of lipolytic enzymes by Lipase_reclassification.

Figure S2. Characterization of hp2-1 using InterPro. Please note that the signal peptide sequence was excluded prior to the characterization. Zn binding site (S52 and D54) and ligand binding site were predicted (A84-T85-S86, S104-I105-H106, N161, D210, and E235).

Figure S3. Signal peptide prediction of hp2-1 using SignalP 6.0.

Figure S4. Effect of PMSF on hp2-1 activity. Error bars represent the standard deviation from technically independent replicates (n = 3).

**Figure S5. Predicted structure of hp2-1 using AlphaFold3.** The structure is colored based on the pLDDT score, which indicates model confidence as follows; Blue: Very high (pLDDT ≥ 90), Light Blue: High (70 ≤ pLDDT < 90), Yellow: Low (50 ≤ pLDDT < 70), Orange: Very low (pLDDT < 50).

**Figure S6. Differential scanning fluorimetry (DSF).** As a control, Bovine Serum Albumin (BSA) at 0.001 mg ml^-1^ in 50 mM sodium phosphate (pH 7.0) containing 62.5 mM NaCl was used. BSA exhibited a clear transition with a major peak around 60 °C, consistent with its reported melting temperature (Jiang et al. 2019).

**Figure S7. Phylogenetic analysis of SemiBin_309 at the order level based on concatenated phylogenetic marker genes of GTDBtk v.2.3.2.** The GTDB species representatives belonging to family Oscillospiraceae and are SemiBin_309 are respectively highlighted in yellow and green for clarity.

**Table S1. Structure comparison using PDBeFold.**

| **##** | **RMSD** | **Nalign** | **Ng** | **%seq** | **Query** | **Target (PDB entry)** | | | |
| --- | --- | --- | --- | --- | --- | --- | --- | --- | --- |
|  |  |  |  |  | **%sse** | **Match** | **%sse** | **Nres** | **Title** |
| **1** | **1.09** | **284** | **3** | **39** | **100** | **6hni:A** | **96** | **296** | **THE LIGAND-BOUND, CLOSED STRUCTURE OF CD0873, A SUBSTRATE BINDING PROTEIN WITH ADHESIVE PROPERTIES FROM CLOSTRIDIUM DIFFICILE.** |
| **2** | **1.23** | **287** | **4** | **33** | **100** | **3lkv:A** | **96** | **297** | **CRYSTAL STRUCTURE OF CONSERVED DOMAIN PROTEIN FROM VIBRIO CHOLERAE O1 BIOVAR ELTOR STR. N16961** |
| **3** | **1.24** | **285** | **5** | **34** | **100** | **3lft:A** | **88** | **296** | **THE CRYSTAL STRUCTURE OF THE ABC DOMAIN IN COMPLEX WITH L-TRP FROM STREPTOCOCCUS PNEUMONIA TO 1.35A** |
| **4** | **1.17** | **276** | **5** | **35** | **100** | **3lft:B** | **88** | **289** | **THE CRYSTAL STRUCTURE OF THE ABC DOMAIN IN COMPLEX WITH L-TRP FROM STREPTOCOCCUS PNEUMONIA TO 1.35A** |
| **5** | **2.49** | **233** | **13** | **12** | **74** | **4zjp:A** | **85** | **270** | **STRUCTURE OF AN ABC-TRANSPORTER SOLUTE BINDING PROTEIN (SBP_IPR025997) FROM ACTINOBACILLUS SUCCINOGENES (ASUC_0197, TARGET EFI-511067) WITH BOUND BETA-D-RIBOPYRANOSE** |
| **6** | **2.45** | **235** | **18** | **17** | **74** | **6gq0:A** | **81** | **280** | **CRYSTAL STRUCTURE OF GANP, A GLUCOSE-GALACTOSE BINDING PROTEIN FROM GEOBACILLUS STEAROTHERMOPHILUS** |
| **7** | **2.4** | **226** | **16** | **12** | **74** | **3huu:A** | **81** | **265** | **CRYSTAL STRUCTURE OF TRANSCRIPTION REGULATOR LIKE PROTEIN FROM STAPHYLOCOCCUS HAEMOLYTICUS** |
| **8** | **2.3** | **220** | **13** | **11** | **74** | **3egc:E** | **89** | **261** | **CRYSTAL STRUCTURE OF A PUTATIVE RIBOSE OPERON REPRESSOR FROM BURKHOLDERIA THAILANDENSIS** |
| **9** | **2.48** | **231** | **13** | **10** | **74** | **1dbp:A** | **85** | **271** | **IDENTICAL MUTATIONS AT CORRESPONDING POSITIONS IN TWO HOMOLOGOUS PROTEINS WITH NON-IDENTICAL EFFECTS** |
| **10** | **2.44** | **226** | **17** | **12** | **74** | **3jy6:A** | **85** | **263** | **CRYSTAL STRUCTURE OF LACI TRANSCRIPTIONAL REGULATOR FROM LACTOBACILLUS BREVIS** |

**Reference**

Jiang B, Jain A, Lu Y, Hoag SW (2019) Probing Thermal Stability of Proteins with Temperature Scanning Viscometer. Mol Pharmaceutics 16:3687–3693. https://doi.org/10.1021/acs.molpharmaceut.9b00598
